# Cholera as a ‘sanitary test’ of British cities, 1831–1866

**DOI:** 10.1080/1081602X.2018.1525755

**Published:** 2018-11-03

**Authors:** Romola Jane Davenport, Max Satchell, Leigh Matthew William Shaw-Taylor

**Affiliations:** a Cambridge Group for the History of Population and Social Structure, Department of Geography, University of Cambridge, Cambridge, UK; b Cambridge Group for the History of Population and Social Structure, Faculty of History, University of Cambridge, Cambridge, UK

**Keywords:** Cholera, industrial revolution, water and sanitation, waterborne diseases, faecal-oral transmission

## Abstract

The malign contribution of northern industrial cities to the stagnation of national life expectancy over the period 1820–1870 forms part of one of the most long-running debates in English economic history, regarding the impact of early industrialisation on living standards. The deteriorating quality of urban water supplies often features in these arguments as the key driver of worsening mortality in this period. Here we use mortality reported from cholera in the epidemic years 1831–1832 and 1848–1849 as an indicator of the extent of sewage contamination of water in English and Welsh towns in this period. Surprisingly, the geography of reported mortality did not indicate that northern manufacturing and industrial towns were especially deficient in this respect. However, logistic regression analyses identified a number of risk factors for high cholera mortality, including location on coal-bearing strata, which was a feature of many industrial towns. Notably, however, textile-manufacturing towns, although often located in coal-rich districts, were associated with low levels of cholera mortality, and high population growth rates did not influence the risk of cholera. Reductions in cholera mortality after 1849 raise the possibility of widespread improvements in water quality after mid-century, rather earlier than is often assumed. However, in contrast to cholera, infant and diarrhoeal mortality remained high especially in northern towns until at least 1900. Several lines of evidence suggest that infants were relatively protected from waterborne diseases such as cholera and typhoid, and therefore did not benefit greatly from improvements in water quality. We conclude (1) that any worsening of water quality in urban areas c.1800–1850 was not confined to new͛ or rapidly growing industrial or manufacturing towns; and (2) infants probably rarely drank untreated water, so high infant or diarrhoeal mortality rates should not be read as indicators of poor water quality, in the English context.

## 1. Introduction

The period of the classic Industrial Revolution, roughly 1760–1850, witnessed unprecedented growth and urbanisation of the British population, and the transformation of the urban hierarchy as ‘new’ industrial and manufacturing towns eclipsed older provincial and county towns. These ‘new’ towns were predominantly located in the northern and midland counties of England, although some port and spa towns in the south also experienced explosive growth.

The impact of such unprecedented rates of urbanisation on life expectancies remains poorly understood. Despite very large gradients in rural: urban mortality rates, life expectancy improved at the aggregate national level between 1750 and 1820, and survival rates of infants and young children rose dramatically in London and probably in other urban centres over the same period (Davenport, 2015; Landers, 1993, Chapters 4–5; Wrigley, Davies, Oeppen, & Schofield, 1997, p. 272). By 1780 many English towns were characterised by annual surpluses of baptisms over burials, in contrast to the preceding ‘urban graveyard’ period, when most towns appear to have functioned as demographic sinks, reliant on in-migration to sustain numbers.

After 1820, trends in life expectancy appear to have become less favourable. At the national level life expectancy stagnated between 1820 and 1870, before resuming a secular improvement that has continued to the present. Trends in urban populations are less clear, for several reasons. First, the quality and representativeness of Anglican parish registers declined progressively after c.1750, and the effects were greatest in urban populations (Krause, 1965). Secondly, although the situation improved enormously with the inception of civil registration of births and deaths in 1837, the data published by the newly created office of the Registrar-General did not provide readily usable sources for calculating urban life expectancies, or for determining the causes of mortality in specific towns (Galley, 1998, Chapter 7).

**Figure 1.**
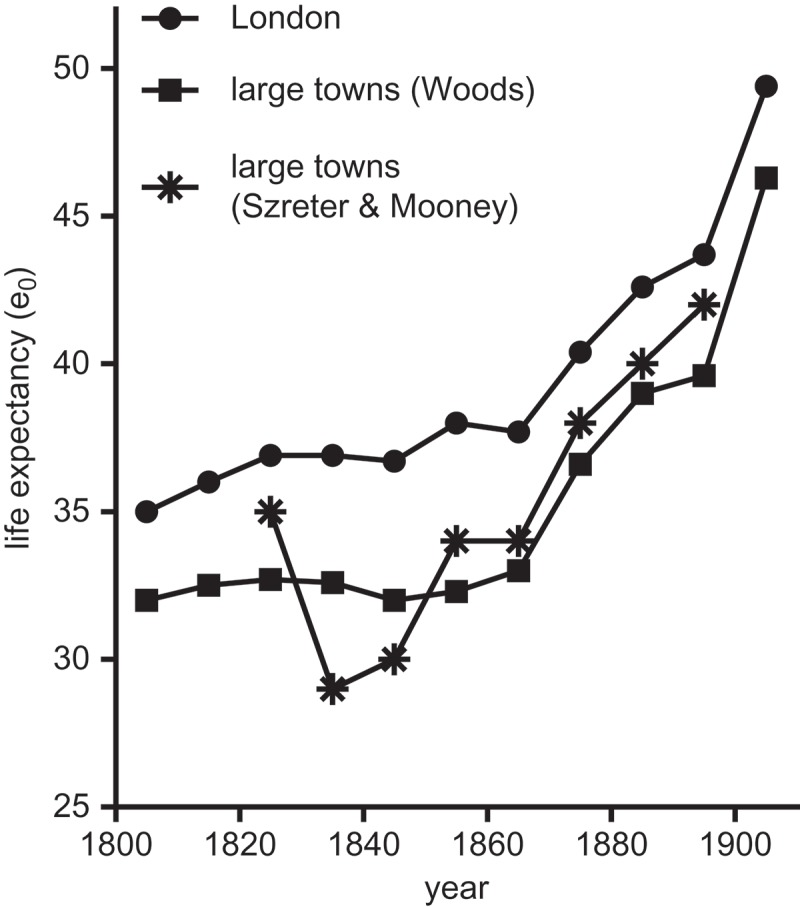
Estimates of urban life expectancies at birth in England and Wales. *Source*: (Woods, 2000, p. 369).

Using a mixture of scant empirical evidence and extrapolation, Szreter and Mooney (1998) argued influentially that life expectancy fell in the national population between 1820 and 1840 as a consequence of worsening conditions especially in northern industrial and manufacturing cities (Figure 1). Szreter and Mooney acknowledged the improvements in mortality that accompanied the rapid growth of population and urbanisation in the first phase of the classic Industrial Revolution period (c.1760–1820). However they argued that the period c.1820–c.1850 was characterised by a new phase of political discord brought on by the cumulative effects of rapid economic growth. In this new atmosphere, according to Szreter, the laissez-faire attitude towards governance that he argued typified the newer administrative structures of northern towns failed to provide much of the basic infrastructure that older towns had through long experience acquired. Szreter went so far as to generalise from the British experience to argue that rapid economic growth inevitably produced disruption and deprivation, with potentially negative consequences for health (Szreter, 1997, p. 715).

Although Szreter and Mooney argued that size and rate of growth alone were not the main determinants of living standards in towns, their conclusions largely support this impression (Floud, Fogel, Harris, & Hong, 2011, p. 232). They concluded that mortality deteriorated sharply in the period c.1820–1850 in northern and industrial towns, and that life expectancy was higher in large southern cities, and probably in the slower-growing and older towns, ‘especially in the south’ (Szreter & Mooney, 1998, p. 107; Szreter, 1997, pp. 700–701). Szreter focussed particularly on the quality of water supplies to British cities in the period c.1800–1870, arguing that although technologies for adequate water provision and sewerage were well known by the 1840s, and water supplies increased after c.1840, the motivation for water supply was principally commercial, rather than to improve population health, and water quality only improved markedly with the progress of municipalisation of supplies from the 1870s (Szreter, 1997, pp. 707–708).

Woods took a more sanguine view of demographic developments in the early nineteenth century. Arguing initially that there was no decline in life expectancy between 1820 and 1860, he later acknowledged the likelihood of some modest decline at the national level (Figure 1; Woods, 1985; Woods, 2000, p. 365, 368). However, he argued that what worsening did occur could be explained by the inevitable epidemiological consequences of urbanisation. These included the massive redistribution of population from relatively low mortality rural areas to higher mortality urban environments, and the more rapid circulation of infectious diseases that occurred as towns grew and surpassed various epidemiological thresholds. He also invoked an autonomous rise in the virulence of scarlet fever. Woods did not accept that local policies were the cause of any increases in mortality in the first half of the nineteenth century, and attributed excess mortality in certain northern towns to the poverty of Irish immigrants (Woods, 2000, pp. 370–371).

The period under debate is one for which we have very little evidence. Szreter and Mooney based their pessimistic assessment of mortality trends in urban centres on evidence from the Glasgow bills of mortality, Carlisle and nine industrialising parishes in Lancashire and the west midlands. Woods instead adopted a modelling approach to decompose national life expectancies into component life expectancies associated with the proportions of the population in different settlement types (Woods, 2000, pp. 363–374). The divergent results of these exercises are shown in Figure 1.

The lack of demographic evidence for the early nineteenth century cannot unfortunately be compensated by evidence regarding sanitary conditions in towns. Although the health of urban populations became an increasingly pressing public health and political issue especially from the 1830s and 1840s, and a number of parliamentary reports were published that incorporated details of water provision and sanitary arrangements in a range of towns, there was no systematic collection of data that would make it possible to compare the sources of water or extent of water provision or sewerage in towns for any date before the 1870s (Royal Commission, 1874).

Economic and medical historians have generally concurred that sanitary conditions worsened in British towns during the first half of the nineteenth century as a consequence of overcrowding and inadequate amenities (Floud, Wachter, & Gregory, 1990, pp. 294–300; Hardy, 1993, pp. 157–158; Harris, 2004; Hassan, 1998, p. 16; Smith, 1979, Chapter 4; Williamson, 1990; Wohl, 1984, pp. 3–5). There is less agreement over whether these changes were associated with a decisive downturn in life expectancy for urban dwellers (Floud & Harris, 1997; Harris, 2004; Williamson, 1990, pp. 258–260) and whether town corporations really had the knowledge and the capacity to ameliorate the problems of urban waste disposal and water provision, or were hampered at least in part by the limitations and complexities of existing technologies (Hamlin, 1988; Rosenthal, 2014, p. 227, 231; Wohl, 1984, pp. 104–107).

London provides a key example here with respect to the unintended consequences of early innovations in water and sanitary provision. London was already the largest city in Europe by 1800, with nearly a million inhabitants, fully ten times larger than its nearest English rivals.^1^ Yet despite its already enormous population, historians of London have generally agreed that serious declines in water quality only manifested in the early nineteenth century. Ever-growing population pressure on water supplies led to the digging of shallow wells that were more easily contaminated by seepage from cesspits, and the problems of waste disposal led to the digging of deeper cesspits, that increased the risk of pollution of the water table (Hardy, 1984). However the greatest threats to health appear to have arisen not from these piecemeal acts but from precocious efforts to modernise water supplies and waste disposal.

Water companies proliferated in London in the early nineteenth century, supplying piped water from the city’s principal watercourses, the Thames and the Lea rivers, or from wells and springs. By 1828 water was piped into over 150,000 households, as well as supplying street pumps (Hardy, 1984, p. 252). The relatively copious provision of water to affluent households made it attractive to install flush toilets which flushed sewage into London’s primitive sewers and thence, untreated, into the rivers. Before 1815 household drains were not permitted to connect to London’s sewers, and sewage was stored in cesspits and removed periodically. The lifting of this longstanding ban resulted in the progressive pollution of the Thames and the Lea. Water for domestic consumption was drawn from the river Thames at low tide, when the water was cleanest. However, tidal rivers tended to carry excrement back up the river at high tide and deposit it on the banks, so even where water was drawn above the sewer outlets, contamination often occurred. Indeed, London experienced multiple large-scale outbreaks in each of the four cholera epidemics between 1831 and 1866, and the majority of these were attributable to sewage contamination of piped water supplies (Farr, 1868, pp. xxxi-xxxix; Luckin, 1977; Snow, 1855, pp. 57–81). After 1857 the private water companies supplying London were required to draw any river water from above the tidal reach of the Thames, and to filter it (Hardy, 1984, p. 266). It seems very likely that these measures helped to prevent major outbreaks of waterborne diseases, except, as in the cholera epidemic of 1866, when regulations were breached. However the efficacy of filtration, and the extent of sewage contamination of the rivers, remained controversial until the 1870s, when advances in bacteriology revealed that sand filtration was in fact an efficient biological process for water purification (Luckin, 1986, Chapter 2).

The complexities surrounding water quality even in London, an ancient city with long-established and progressively modified local and metropolitan administrative frameworks, and where piped water provision and sewage disposal were undoubtedly more advanced than anywhere else in England, indicate the difficulties that contemporaries faced in evaluating the best means of providing clean water and waste disposal. A cornerstone of miasmatic theories of disease that dominated medical opinion in the period was to prioritise the removal of waste from urban and domestic spaces. Initially this involved the use of watercourses to dispose of sewage, at the expense of water quality. As Edwin Chadwick, the most influential of nineteenth-century health reformers, argued, the consequences in terms of river pollution were ‘of almost inappreciable [i.e. insignificant] magnitude in comparison with the ill-health occasioned by the constant retention of … pollution in the most densely peopled districts’ (Wohl, 1984, p. 239). The greater availability of piped water for waterborne sewage disposal, coupled with inadequate market and legislative incentives to provide clean sewage treatment, then coincided with medical theory to produce river pollution on an apparently unprecedented scale. In this scenario, towns that were pro-active in adopting newer means of waste disposal could inadvertently have increased the pollution of domestic water sources. This trend could be exacerbated, as it was in London, where these watercourses were increasingly used to provide piped water. Conversely, towns that continued to rely mainly on domestic cesspits and middens for waste disposal, and/or wells or other very localised and diverse sources for water, may have been at lower risk of major outbreaks of waterborne disease. Only when sewage treatment became widespread, and water filtration the norm, which occurred progressively from the 1870s, is it likely that water and sanitary improvements were correlated straightforwardly with improvements in water quality. Thus an historical knowledge of expenditure on water and sanitary works, even if it were possible, would be insufficient to predict water quality and associated health outcomes, in the period before c.1870.

The scanty data regarding mortality rates and water quality in urban centres in the first half of the nineteenth century have made it difficult to distinguish between the pessimistic vision of Szreter and Mooney, and the more sanguine view of Woods. In this paper we attempt to use an alternative source that covers the entire country to evaluate the state of water quality in English towns in the second quarter of the nineteenth century. We then address the related question of whether water quality was a major influence on urban mortality patterns in this period.

### 1.1. Water supplies and faecal-oral transmission of infectious diseases

One of the most important consequences of the aggregation into large and dense populations that accompanies urbanisation is the problem of waste disposal. Urban populations generate large volumes of human and animal excrement, and these often contain pathogens that can cause a range of (mainly) gastrointestinal diseases, spread by faecal-oral transmission routes. Diseases spread by faecal-oral routes include cholera, dysentery, typhoid and a number of other bacterial, protozoan and viral pathogens that cause diarrhoea. Transmission can occur via contamination of water supplies when sewage is dumped in waterways or seeps from latrines into nearby wells or into the water table more generally. Other transmission pathways include transfer during food preparation; direct human to human transfer due to inadequate hand-washing and domestic hygiene; and transfer from faeces to food or skin via insect vectors, especially flies.

While historians often use the term ‘waterborne diseases’ to describe the category of diseases spread via the faecal-oral route, these diseases vary enormously in the extent to which they are spread via water (Van Poppel & van der Heijden, 1997). Ewald (1991) surveyed a range of historical outbreaks where the causal pathogen and the source of the outbreak could be identified (Table 1). Documented transmission pathways included contaminated water sources, direct person-to-person transmission, transfer during food preparation, and contamination of food and drinking vessels. Transmission by insects was not documented, because such transmission has proven very difficult to demonstrate. Ewald concluded that outbreaks of the classic ‘Asiatic’ cholera (*Vibrio cholerae*) were caused largely by ingestion of contaminated water. Typhoid outbreaks were caused by contaminated water or by contamination of food or drink by infected handlers. The *Shigella* species that cause dysentery (for which bloody diarrhoea was the defining symptom) varied substantially in their virulence and reliance on waterborne transmission, with the most lethal form, *S. dysenteriae*, associated mainly with waterborne outbreaks. Outbreaks associated with less virulent pathogens, such as *E. coli* and non-typhoid salmonellas, that are typically associated with food poisoning incidents and with diarrhoea, were very rarely waterborne.

**Table 1.** Pathogens with faecal-oral transmission routes.

Pathogen	Mortality (% of cases)	Waterborne outbreaks (% of all outbreaks)
*Vibrio cholerae*, classical biotype [Asiatic cholera]	15.7	83.3
*Shigella dysenteriae* type 1[dysentery]	7.5	80.0
*Salmonella typhi* [typhoid]	5.8	74.0
*Vibrio cholera*, el tor biotype	1.44	50.0
*Shigella flexneri*	1.32	48.3
*Shigella sonnei*	0.65	27.8
Enterotoxic *E. coli*	<0.1	20.0
*Campylobacter jejuni*	<0.1	10.7
Non-typhoid salmonella	<0.1	1.6

Source: (Ewald, 1991, pp. 83–119)

This diversity of transmission pathways for gastrointestinal diseases has obvious implications for interventions designed to prevent transmission. In attempting to assess the impact of improvements in water supply and sanitation historians have used a number of different outcome measures, including all-cause and infant mortality, infant diarrhoeal mortality, typhoid mortality, and, in the English case, mortality attributed to the category ‘Typhus and Typhoid’. These studies have produced mixed findings, ranging from a halving of typhoid and infant mortality in response to water filtration in U.S. cities, to no effect or even negative health outcomes (Cutler & Miller, 2005; van Poppel & van der Heijden, 1997; see also other articles in this issue). The potential reasons for these conflicting results are very perceptively reviewed by van Poppel and van der Heijden (1997). Here we draw attention briefly to three major sources of potential ambiguity.

First, as discussed above, pathogens differ in their modes of transmission. Therefore the effects of interventions may be overlooked if an outcome measure is used that is not responsive to the intervention. For example, filtering water supplies to reduce faecal contamination would be expected to have a large impact on diseases such as cholera, dysentery and typhoid, where contaminated water is a major source of infection, but possibly little or no impact on diseases transmitted through food handling or by flies (such as may be the case for most causes of diarrhoea in infants). Conversely, the use of middens to dispose of faecal and other waste, a practice apparently widespread in Lancashire in the 1840s (Royal Commission, 1845, pp. 368–370), may have promoted fly-borne and human-to-human transmission of diseases, but may have posed a relatively low risk in terms of water contamination compared with the use of cesspits or water-based disposal of faeces into drains.

The evidence that gastrointestinal diseases differ in their routes of infection and susceptibility to interventions may explain some of the otherwise puzzling differences in the chronology of improvements in mortality caused by different gastrointestinal diseases. For example, in Condran’s in-depth ward by ward study of Philadelphia, typhoid mortality fell rapidly in response to the introduction of water filtration in the early twentieth century, but diarrhoeal mortality was relatively unaffected (Condran, 1987). A similar outcome was reported in one of the earliest surveys of the effects of improvements in sewerage and water supply in selected English towns, which noted strong effects of these works on typhoid mortality over the period c.1845–1865, but equivocal effects on diarrhoeal mortality (Medical Officer of the Privy Council, 1867, p. 35, 43).

**Figure 2.**
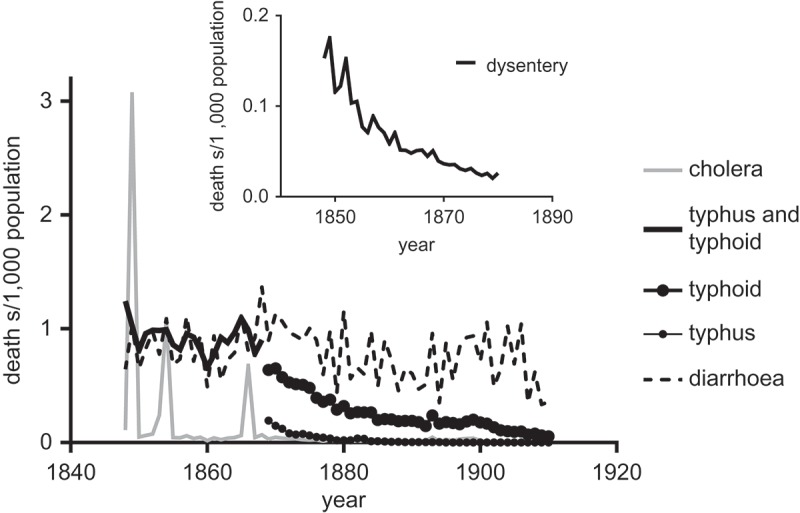
Crude death rates attributed to diseases with faecal-oral transmission pathways, and typhus, England and Wales, 1848–1911. *Notes*: Deaths attributed to dysentery and diarrhoea were reported separately 1848–1880 and in a single category 1881–1911 (when dysentery rates had fallen to negligible levels: see inset).*Sources: Annual Reports of the Registrar-General*, 1849–1911

Similar discrepancies are evident in Figure 2, which presents annual crude death rates for major categories of gastrointestinal diseases over the period 1848–1910, the period for which we first have continuous annual records of causes of death for England and Wales. The cholera epidemics of 1848–9, 1854 and 1866 are clearly visible. Asiatic cholera disappeared from England after 1866, and the cholera deaths reported after 1866 refer to deaths attributed to ‘English’ cholera. These declined sharply across the 1870s. Typhoid was not reported separately from louse-borne typhus in national reports until 1869, despite the very different aetiologies of the two diseases. However from 1869 typhoid mortality was clearly in decline. Dysentery was reported as a separate category in the Registrar-General’s reports until 1880, and declined across the period 1850–1880. In contrast mortality attributed to the category ‘Diarrhoea’ improved to some extent in the 1870s and 1880s before resurging during the hot summers of the 1890s. Diarrhoeal mortality only improved decisively after 1900, together with infant mortality as a whole. The early declines in those diseases that were most dependent on waterborne transmission, cholera, typhoid and dysentery, suggest superficially that improvements in water quality were underway at least by the 1860s (Luckin, 1986, pp. 119–122).^2^ However, it would appear that any such improvements had relatively modest effects on mortality from other gastrointestinal infections such as diarrhoea, where other routes of transmission may be more important.

A second factor affecting the observed relationship between water improvements and health is the quality of the intervention. As we have argued above, even if we had details of the numbers of houses supplied with piped water, or connected to sewerage, we could not treat these data as measures of access to clean water or effective sanitation, because the quality of the water delivered, or effectiveness of sewage disposal, is unknown. In the nineteenth century even where water quality was tested using assays for organic matter or bacteriological content, the results could not be related directly to levels of faecal contamination, because they were insufficiently specific (Hamlin, 1990, pp. 117–121, 299; Luckin, 1986, Chapter 2) and could be dominated by non-pathogenic sources such as algal blooms.

A third key factor is the effect of baseline conditions. Private water companies proliferated in Britain in the early nineteenth century, providing piped water to relatively affluent households in many urban areas, as well as to public pumps in some areas. These new sources of water co-existed alongside longstanding sources including private and public wells, springs (sometimes delivered to public pumps or fountains via conduits), and streams and rivers. It is critical to recognise both that ‘improved’ water sources could be of lower quality than unimproved sources, and that unimproved or ‘traditional’ sources varied enormously in quality. Jaadla and Puur (2016) provided an elegant illustration of the potential range in quality of ‘traditional’ sources of water, in the Estonian city of Tallin at the end of the nineteenth century. Using linked census and church registers together with a contemporary survey of water sources they were able to compare infant mortality in individual households according to water source. Controlling for paternal education and occupation, mother’s age, household size, and waste disposal method, infant mortality was four times higher in households supplied from the river compared with those supplied by artesian wells, and twice that of households supplied from groundwater wells. In this case the introduction of piped water may have conferred little additional benefit on those already using artesian sources, and could have raised mortality if it derived from a less pure source.

### 1.2. Cholera as a test of water purity, 1831–66

Here we examine water quality in English towns in the middle decades of the nineteenth century through the lens afforded by the four cholera epidemics that swept Britain in the period 1831–1866. This is a period for which we have only unsystematic snapshots of water provision in English cities, and no records of typhoid mortality that might serve as proxies for water quality (Figure 2).^3^ However we consider that cholera mortality provides a good test of water quality in towns in this period for three reasons.

Firstly, cholera is pre-eminently a waterborne disease (Table 1). Thus mortality from cholera can be regarded as a measure of the extent to which water supplies were contaminated by excretions from cholera victims, especially where the outbreak exceeded a certain threshold.^4^

Secondly, cholera was a new disease and each epidemic was documented systematically in a series of reports, providing insights into spatially disaggregated disease patterns that are otherwise unavailable in this key period. Asiatic cholera (the classic *Vibrio cholerae* O1 CL biotype: Finkelstein, 1996) erupted out of the Ganges delta in six pandemics in the nineteenth century, first reaching western Europe in 1831. The first cases occurred in Britain in the autumn of 1831, followed by a major epidemic in the summer of 1832. The second epidemic, of 1848–1849 showed a similar pattern, with a ‘herald wave’ in the autumn of 1848 and a national outbreak in the summer of 1849 (Tien, Poinar, Fisman, & Earn, 2011). A third epidemic in 1854 was concentrated in London, and the last major outbreak in Britain was in 1866. Britain was precocious in escaping cholera, and devastating epidemics recurred in the USA and on the continent in the period 1883–1892.

The third useful feature of cholera as a test of water quality is that the cholera biotype implicated in nineteenth-century pandemics was highly lethal, and produced fairly distinctive symptoms in severe cases at least in adults. Although contemporaries considered the case-fatality rate of cholera to be almost 50%, based on medical reports of cases and deaths, it is likely that the true case-fatality rate was rather lower because not all cases of infection were detected or reported. Ewald estimated an average case-fatality rate for classical *Vibrio cholerae* of c.16 %, somewhat lower than contemporary estimates, but nonetheless very lethal (Table 1). Importantly, cholera was virulent enough to kill young adults as well as children and the elderly, and to kill the well nourished as well as the poor. Most reports of cholera outbreaks documented only deaths from the disease, rather than cases, although diarrhoeal deaths were also noted. High lethality is a useful property of the disease from a historical vantage point, because it makes mortality statistics a relatively useful guide to the incidence of the disease and the size of the outbreak.

**Figure 3.**
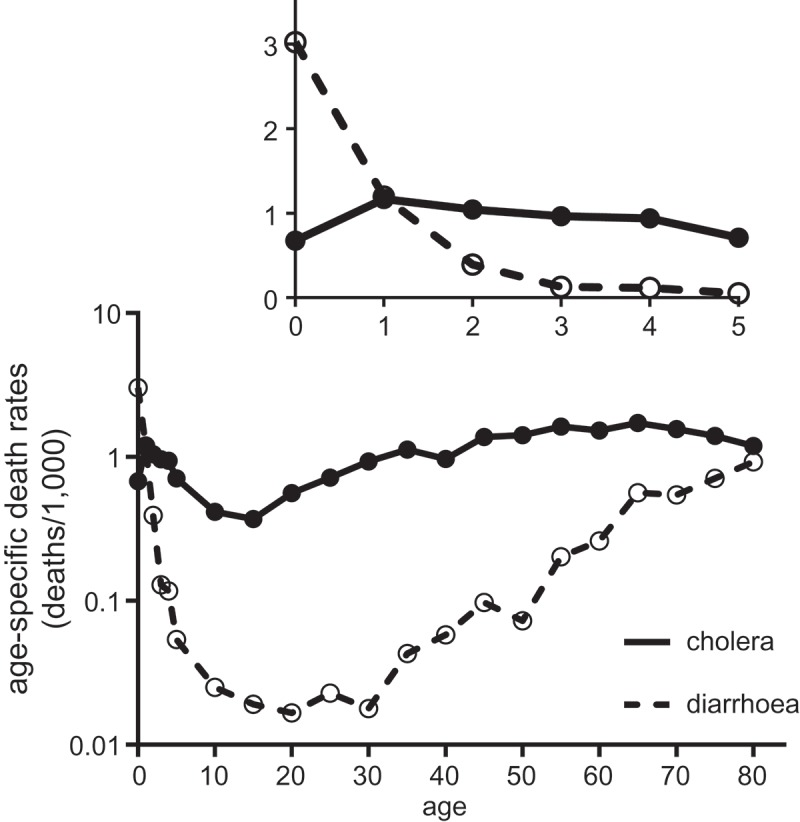
Age-specific mortality attributed to cholera and to diarrhoea, males, England and Wales, 1849. *Source*: Davenport (2007).

The high lethality of cholera is evident both in the absolute mortality rates, and in the age pattern of mortality (Figure 3). In contrast to reported diarrhoeal mortality, which followed the J-shape typical of all-cause mortality and of diarrhoeal mortality in a wide range of populations (Preston, 1976, pp. 98–100), cholera did not spare even young adults, and the curve of cholera mortality with age was relatively shallow (for a similar pattern in 1832 see Morris, 1976, pp. 82–83 and his Figure 1). The high lethality of cholera to adults was, together with its Asiatic origins, a major source of the panic caused by the disease. As Morris (1976, pp. 12–14) noted, total cholera mortality was unremarkable compared with the annual toll of diarrhoeal disease in infants, and the strictly demographic impact of cholera in Britain was negligible.^5^

The age-specific patterns of mortality in Figure 3 (inset) also raise questions regarding detection of the disease. The rates reported imply that mortality from cholera was lower in infancy than later in childhood, a reversal of the usual pattern of gastro-intestinal infection as indicated by diarrhoeal deaths, where mortality is concentrated at the extremes of age. The obvious explanation is that cholera was so lethal in infants that it killed them before the characteristic symptoms, of blue pallor and sunken eyes in particular, could develop. We discuss this possibility later in the paper.

While cholera epidemics have high potential to reveal the vulnerability of water sources to faecal contamination, there are also several potential problems with their use for this purpose. First, as a new disease the spread of cholera within the British population depended on transmission between settlements, and therefore on patterns of movement. While we wanted to use cholera mortality as an indication of water quality, the occurrence of an outbreak was predicated on the successful introduction of cholera into the community, a probabilistic event related to the size of the population and the extent of connectedness with other populations. Therefore we attempted in our analyses to distinguish between the risk of cholera import, which could be considered dependent on the size and connectedness of populations, and the risk that a large outbreak occurred. However it was possible that places with very high rates of contact with other populations could report relatively high numbers of deaths attributable to imported cases, even in the absence of any contamination of local water sources.

**Figure 4.**
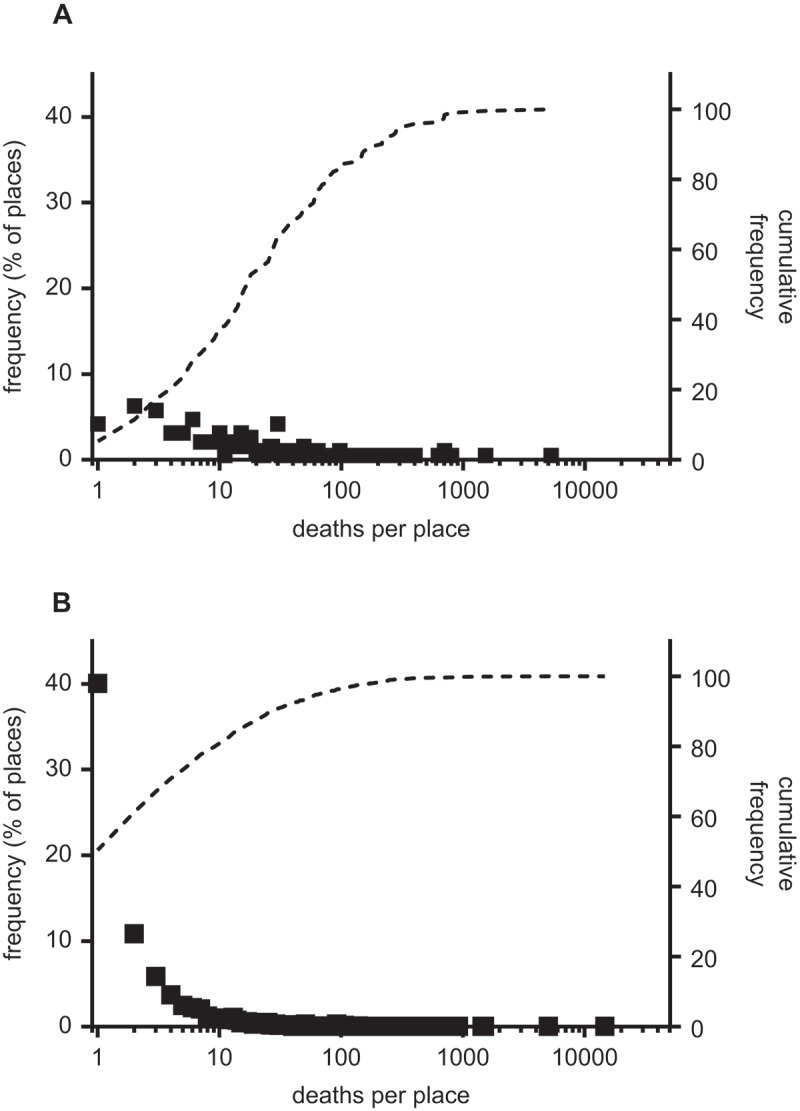
Frequency distribution (squares) and cumulative frequency of cholera deaths per place in 1831–2 (a) and 1848–9 (b). *Sources*: *Manuscript as to the incidence of cholera in Great Britain (TNA, PC1/108)*, Baly and Gull (1854), Farr (1852), Sutherland (1850).

A second problem is that, as discussed above, in most cases only deaths from cholera were recorded, not cases, and these were probably under-reported. Therefore outbreaks where cases occurred without deaths, or where cholera deaths were not recognised, went unrecorded, thereby distorting our picture of the spread of the disease. This problem is most serious with respect to the 1831–1832 epidemic. This epidemic pre-dated the development of the civil registration system, and the only report providing counts of deaths by place for the country as a whole consists of a hand-written list of outbreaks.^6^Figure 4 plots the distribution of reported outbreaks in 1832 and 1849. In 1849 the frequency of cholera deaths per place followed a roughly exponential distribution, with over half of places reporting only one or two deaths (Figure 4(b)). This pattern was consistent with the widespread dissemination of cholera, and the fact that deaths were only the tip of the iceberg of infectious cases. However in 1832, although the 1832 report was unusual in reporting cases as well as deaths, only two places reported cases without deaths, and the distribution of reported outbreaks was clearly skewed towards larger outbreaks. In 1831/2 very few doctors had encountered cholera before (with the exception of those who had practised in India), and it is very likely that small outbreaks in particular went unrecorded. In addition, the machinery of reporting that was established from 1837 was absent in 1832, and the report was compiled on the basis of voluntary reports to the Board of Health (Durey, 1979, pp. 42–43; Farr, 1868, p. xlv). It seems likely therefore that for 1832 the reported cholera deaths under-represented the number of outbreaks, and probably also undercounted the numbers of deaths attributable to cholera even in outbreaks that were recorded.

## 2. Data sources and methodology

In this paper we focus on the first two cholera epidemics in England and Wales, in 1832 and 1849. These outbreaks occurred before the recognition of the waterborne nature of the cholera pathogen, and in the period where urban water supplies are argued to have reached a nadir with respect to both quality and quantity. Therefore we assume that attempts to prevent the spread of the disease were relatively ineffectual (although isolation of victims was practised to some extent, and is very likely to have reduced transmission, depending on the methods of disposal of contaminated wastes). It is also the case that these are the only two epidemics for which we can derive crude death rates from cholera for urban populations.

For the 1831–1832 epidemic the main source of quantitative evidence for epidemic cholera mortality is a report compiled by the Board of Health consisting of a list of places with totals of cholera cases and deaths, dates of first and last cases, and census populations in 1831.^7^ For subsequent epidemics our main sources were the official reports published by the Registrar-General (for 1849, 1854 and 1866), reports of the General Board of Health (for 1848/9: Sutherland, 1850), and the Royal College of Physicians (for 1849: Baly & Gull, 1854). The 1848–1849 epidemic was the most thoroughly documented, although no single report covered the epidemic in its entirety (the Registrar-General’s report for instance only reported deaths occurring in 1849 in sufficient spatial detail, so it was necessary to extract information on the ‘herald wave’ in 1848 from Baly & Gull, 1854; and Sutherland, 1850). In the case of the 1854 epidemic the Registrar-General produced an official report that covered only London in detail, and we have not attempted to reconstruct this epidemic.

The most comprehensive reports, those of the Registrar-General for 1849 and 1866, reported deaths from cholera and diarrhoea by registration sub-district (‘RSD’) (Farr, 1852, 1868). These RSDs were the lowest units in which the Registrar-General usually reported mortality, and were fairly disaggregated, with c. 2,000 units for England and Wales. However the RSDs were based on poor law unions and were designed to combine where possible urban and rural settlements, making them unsuitable for the measurement of mortality specifically in urban areas. Fortunately the Registrar-General’s reports for 1849 and 1866 included excerpts of the individual registrars’ reports for each RSD, and these gave the specific location and date of the first and usually last cholera deaths in the district, and often included other information on the geographical distribution of deaths within the RSD. This made it possible to document the chronology of the spatial spread of the disease. For 1849 the local registrars also reported totals of cholera deaths for a large number of individual places including most affected towns, making it possible to calculate crude death rates for the towns. In 1866 affected settlements were noted individually, but the numbers of deaths in each were not given, so we could not measure cholera mortality by settlement. Because we were concerned to measure cholera mortality specifically in towns we extracted all information on cholera and diarrhoeal deaths in individual settlements in 1848–1849.

In order to measure mortality we converted counts of cholera deaths to crude cholera death rates for towns using these totals and urban population totals for 1851 (collated by Bennett, 2012).^8^ We did not create death rates for RSDs, because in this case the denominator generally referred to a much larger population than the population affected by cholera. For the 1832 epidemic we used the deaths and town populations reported in the original source to calculate crude mortality rates, and town populations for 1831 from Bennett (2012) for places with no reported deaths. For towns where no cholera deaths were reported then we assumed that the cholera death rate was zero, although as noted above this was probably not the case in 1832. We calculated urban growth rates for the periods 1811–1831 and 1811–1851. We used 1811 populations as the base for these calculations because more data were available for 1811, especially for small towns, than at other dates (Bennett, 2012).^9^

In order to test whether there was a relationship between the size or growth rate of urban settlements and cholera mortality we adopted a regression modelling approach. A standard approach to modelling counts (in this case cholera deaths) is to use regression models that assume a Poisson or negative binomial distribution of outcomes. However our counts data were characterised by severe over-dispersion and were not amenable to this type of modelling (Figure 4). Instead we created a binary variable that split our sample into towns with modest or no cholera mortality, and those with higher cholera mortality, and modelled the probability of high cholera mortality using logistic regression models. In order to reduce the problems associated with small numbers and integer counts,^10^ we considered only towns with populations of 5,000 or more in 1851, for which we had complete geographical and other data (n = 307). We considered each explanatory variable separately and by stepwise addition to multivariate models, and retained those that were significantly associated with cholera mortality (*p* < 0.05) and/or improved the fit of the model, as assessed by likelihood ratio tests of nested models. The threshold for designating a town as having high cholera mortality was set at a crude cholera death rate of ≥ 1/1,000. Sensitivity analyses exploring a range of thresholds between 0.5 and 3/1,000 indicated that the models were not very sensitive to the threshold used, in the case of 1849. However in the case of the 1832 epidemic the bias in reported deaths towards large outbreaks made our models very sensitive to the choice of the binary distinction between high and low mortality. Therefore our analysis of the 1832 epidemic is confined to descriptive statistics.

In order to control for co-variates that might have influenced the relationship between cholera mortality and population size or growth, we created variables that measured connectedness to other populations (to proxy the degree of import of cholera cases), and geographical features that affected local water supplies. To create these variables we needed some measure of the geographical extent of each town, to relate to geological, hydrological and transport network features. For this purpose we used a set of ‘urban footprints’, polygons that were drawn to define the built-up area of every English town c.1890. The footprints related to c.1890 because this was the first decade in which the Ordinance Survey produced high resolution maps that covered the whole country for a well-defined and comparable time period. These town footprints therefore over-estimate in many cases the physical extent of towns in 1832–66, but are currently the only means of defining the physical extent of urban settlements, both for small towns entirely within a single parish, and for larger towns that comprised multiple parishes and parts of parishes. The extent of transport networks was determined from a dynamic GIS of the transport network of England and Wales, which provided information on the operational network in the year of each cholera outbreak (Henneberg, Satchell, You, Shaw-Taylor, & Wrigley, 2017; Rosevear et al., 2017; Satchell, Newton, & Shaw-Taylor, 2017). We defined towns as connected to a navigable waterway (river or canal), railway or turnpike road if the town footprint was within 500 metres of one or more of these transport features (defined as a rail station, for railways).^11^ Ports were identified from a ports directory of 1843 (Daniel, 1843).

To characterise potential influences on local water supply and quality we created variables that indicated whether a town was located on a river (tidal or inland: Ordnance Survey, 2016), and what proportion of the town footprint was potentially liable to flooding (Environment Agency, 2015). To test whether elevation or hilliness was important in facilitating drainage or accessing more local and unpolluted sources of water we created variables encoding the mean and standard deviation (a measure of ruggedness) of altitude (variables represented logged values, to normalise the distributions of values) (European Environment Agency, 2015). In an attempt to capture some aspects of the quality of locally available water supplies we used a low-resolution hydrogeological dataset that classified the underlying bedrock into seven categories, associated with four types of aquifer (high, moderate and low productivity, and essentially no groundwater) (British Geological Survey, 2017). We calculated the proportion of each town footprint associated with each rock or groundwater type. The distributions of these values were highly non-normal, and so we constructed binary variables indicating whether each rock or aquifer type comprised 50% or more of the urban area. Approximately 60 % of town footprints consisted of 90% or more of a single bedrock or aquifer type.

Northern textile towns (‘mill towns’) and mining towns comprised some of the fastest-growing towns in the early nineteenth century, and embodied many of the characteristics Szreter associated with rapid disruptive economic growth. To test whether these types of towns were associated with high cholera mortality we created binary variables that indicated a significant presence of mining and textiles, based on occupations reported for adult males by town in the 1841 census. Towns were considered textile towns if 10% or more of adult males were employed in textile manufacturing, and mining towns if 5 % or more were employed in mining (Southall et al., 2004).^12^

Data were mapped in Arcmap 10.5 (ESRI, 2017) and analysed using Stata 14.0 statistical software (StataCorp, 2015).

## 3. The geography of cholera mortality in the nineteenth century

**Figure 5.**
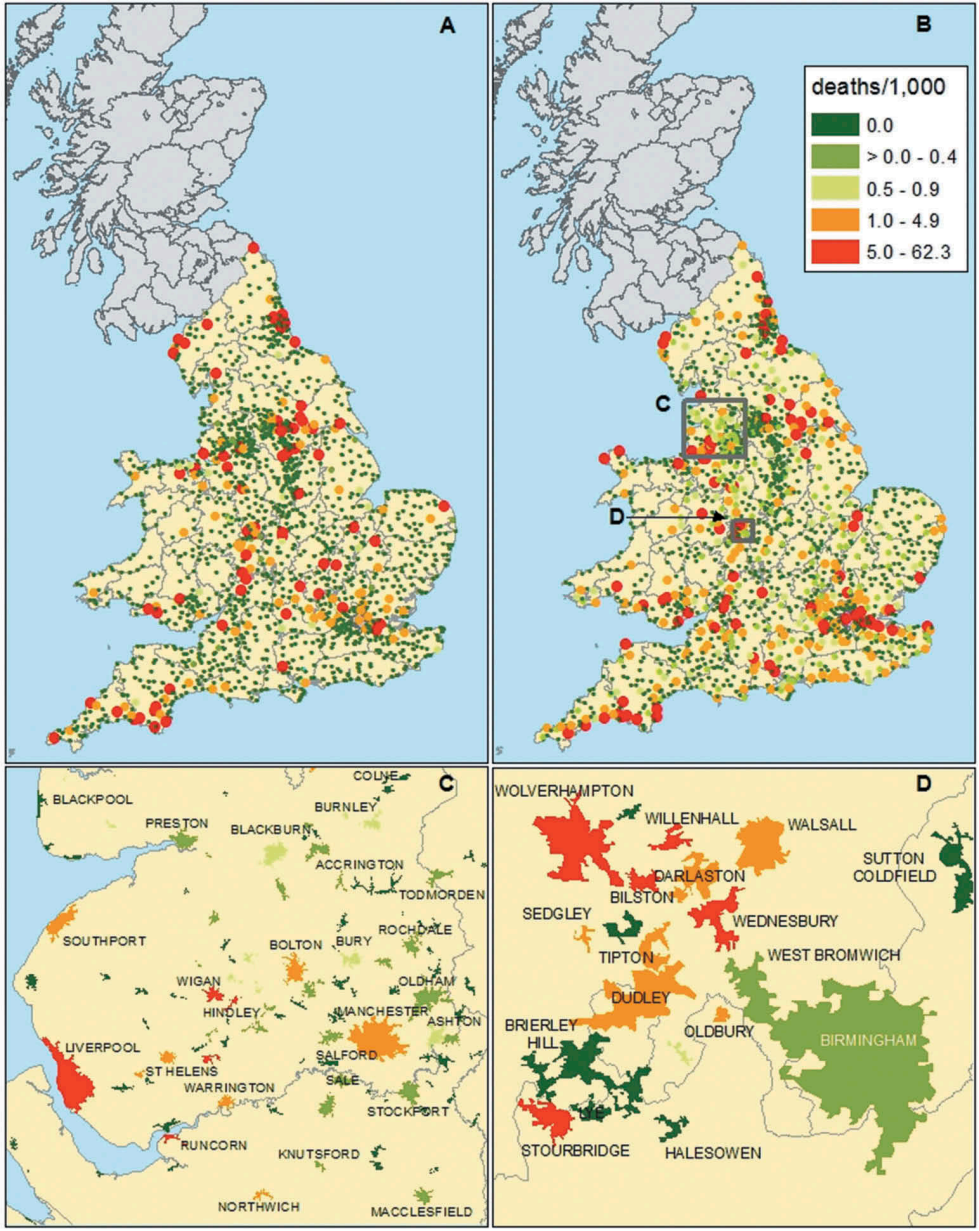
Crude cholera mortality in towns, 1831–2 (a) and 1848–9 (b–d). *Sources*: see Figure 4.

**Figure 6.**
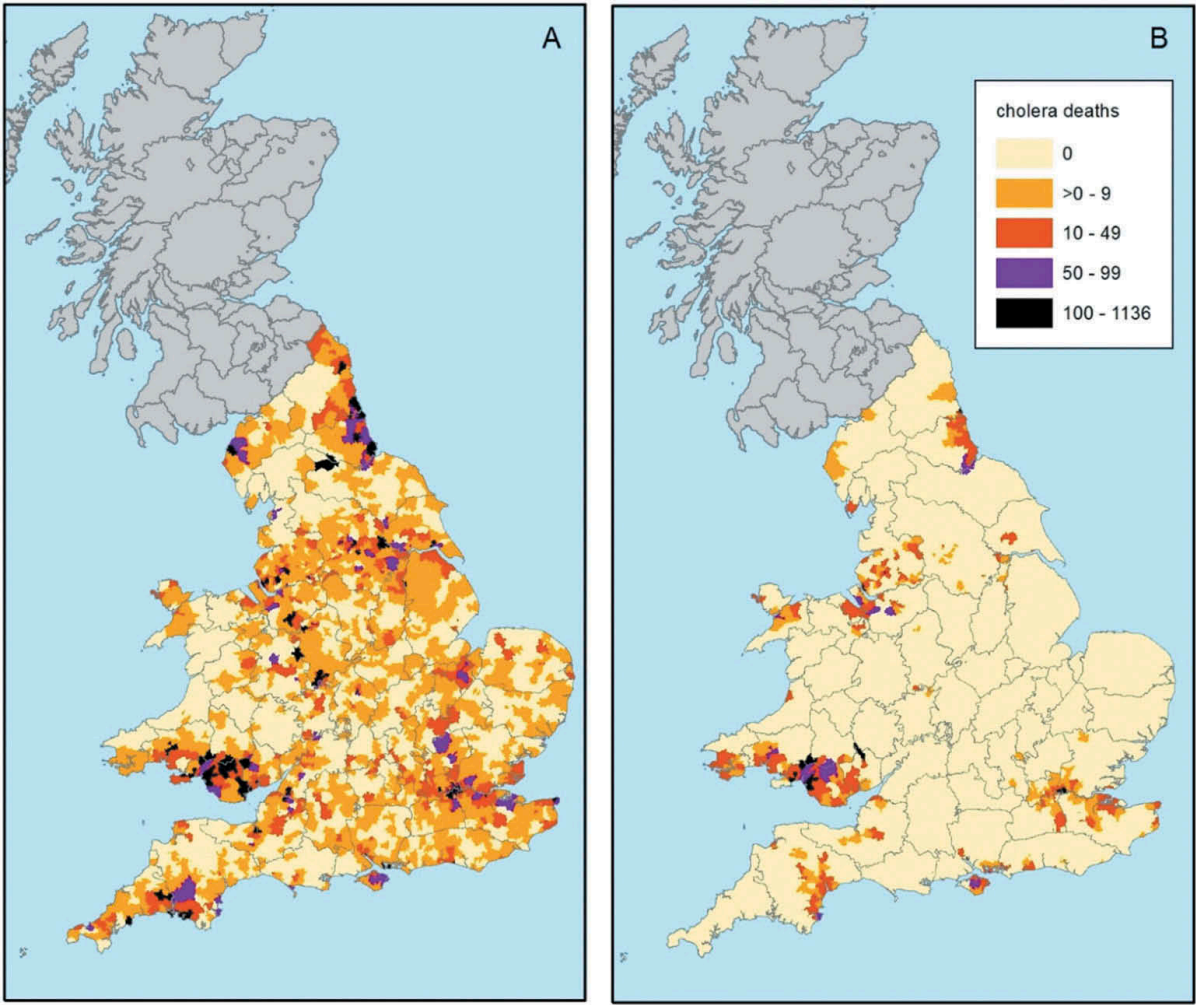
cholera deaths in registration sub-districts, 1848–1849 and 1866. *Sources*: see Figure 4; Farr (1868).

Cholera invaded Britain four times (in 1831/2, 1848/9, 1854 and 1866), however after 1849 mortality was focussed on London (Table 2). The geographical distribution of cholera in 1832, 1849 and 1866 is mapped in Figures 5 and 6. In the case of the 1831/2 epidemic the towns with non-zero death rates probably represent mainly those places with especially severe outbreaks, as explained above. Nonetheless it is clear that cholera was widely dispersed throughout Britain. For the 1848–1849 epidemic the data are displayed as crude mortality rates for towns (Figure 5b–d) and as deaths by registration sub-district (Figure 6(a)). These maps illustrate the very widespread distribution of cholera in 1848/9. By 1866 although mortality was dramatically reduced relative to previous epidemics, cholera deaths still occurred in widely dispersed districts, indicating the limited success of international quarantines (Figure 6(b)).^13^ However in 1866 only the Welsh coal fields and East London suffered mortality rates comparable to those of previous epidemics.

**Table 2.** Cholera deaths reported by epidemic.

epidemic	England and Wales	London	Scotland
1831–32	21,882	5,275	9,592
1848–49	53,293	14,137	6,857
1853–54	20,097	10,738	6,848
1866	14,378	5,596	1,270

Source: (Creighton, 1894, p. 816, 821).

**Figure 7.**
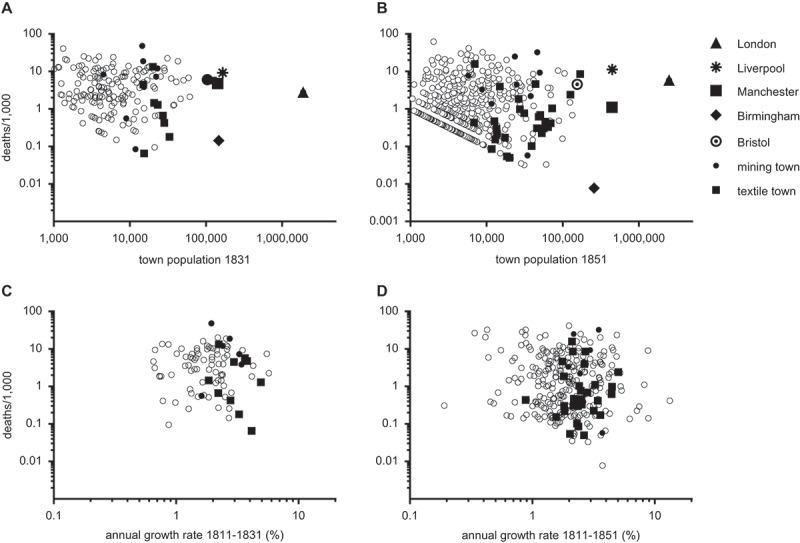
Crude cholera mortality (cholera deaths per 1,000 population) by population of towns or population growth rates in 1831–1832 (panels a,c) and 1848–1849 (panels b,d). *Notes*: Data in panels c,d refer to towns of population 5,000 or more in either 1831 or 1851. Data are plotted on log-log axes.*Sources*: see Figure 4.

Figure 5(b) focuses on urban mortality in the 1848/9 epidemic. The insets (Figure 5(c,d)) map crude cholera mortality by ‘town footprints’, for Lancashire and for the coal-mining ‘Black Country’ adjacent to Birmingham. With the exception of Liverpool, most of the manufacturing towns of Lancashire experienced relatively light cholera mortality. The predominantly textile towns located in the western foothills of the Pennine hills, such as Ashton under Lyne, Blackburn, Burnley, Oldham and Rochdale, were especially fortunate. Remarkably, Birmingham almost entirely escaped cholera outbreaks in each of the four epidemics, and Birmingham experienced the lowest crude cholera mortality in our sample of towns with population 5,000 or more in 1849, despite a census population of over 250,000 in 1851, and its role as a transport hub wide open to disease importation (Figures 5(d), 7(b)). This low mortality was despite a relatively scarce piped water supply, a neglect that may be explained partly by Birmingham’s natural geographical advantage of abundant supplies of spring water derived from the red sandstone beneath the town (Durey, 1979, p. 44; Royal Commission, 1845, p. 87). In stark contrast to Birmingham the mining towns and settlements of the adjacent Black Country suffered some of the worst cholera mortality, with Bilston almost topping the crude death toll in 1832.

Figure 7(a,b) plot crude cholera mortality in the first two cholera epidemics, for towns, by their populations in the nearest census year (1831 or 1851). Both axes are logged, to display plot values clearly. Notably, the most serious outbreaks, on a per capita basis, occurred in small towns. While the highest cholera mortality reported in large towns (100,000+ population) was 11.3 deaths/1,000 in Liverpool in 1849, 37 smaller towns exceeded this rate, of which only the mining towns of Merthyr Tydfil and Bilston had populations over 20,000. The highest rates reported were 62.3/1,000 in Mevagissey (Cornwall, population 2,022 in 1851), and 41.4/1,1000 in Neath (Glamorganshire, population 5,841). Durey argued that a major reason for this pattern was probably that the diversity of water sources increased with the size of the population (Durey, 1979, pp. 38–47). Small towns might rely on a single stream or a small number of wells, and therefore contamination of one source affected a high proportion of the population. Conversely, larger towns generally depended on a greater number of individual water sources, and so outbreaks were usually localised and affected only a small proportion of the population (although this obviously varied depending on the sources of water, and the proportion of houses that were supplied by piped water from a single source). Where a large population depended on a large number of individual wells, as in Birmingham, then the statistical consequences of contamination of any given water source were small. However in London the relatively heavy reliance on piped water sources and the diversity of water suppliers meant that ‘localised’ outbreaks could affect thousands of households when a water source was contaminated. As a consequence, London recorded relatively high cholera mortality in every epidemic. Liverpool was unusual in experiencing relatively widespread outbreaks affecting a large proportion of the city, and was also distinguished by high mortality in every epidemic, although mortality declined substantially after the 1849 outbreak (Durey, 1979, p. 41; Kearns, Laxton, & Campbell, 1993). Figure 7 also highlights the relatively high mortality of Bristol, and more modest mortality of Manchester (then England’s third city, and notorious for its urban slums). Mining towns experienced some of the highest per capita cholera rates, whereas textile towns generally experienced much lower rates.

Figure 7(c,d) plot crude cholera mortality in towns by the annual growth rate of the population since 1811. The lack of any apparent relationship between population growth rates and cholera mortality in either 1831–1832 or 1848–1849 did not support the proposition that rapidly growing towns were at especial risk of large cholera outbreaks. However it was possible that other factors affecting risk of cholera, such as risk of import or adverse geography, were masking the relationships between population size or growth, and cholera mortality.

To test whether there was an underlying relationship between urbanisation rates and cholera mortality we performed a logistical regression analysis of the 1849 epidemic. Table 3 presents results from bivariate analyses comparing the pairwise relationships between high cholera mortality and each explanatory variable in 1848–1849, and the results of multivariate models that included transport connectedness, topographic and economic variables. The multivariate models include only those explanatory variables that improved the fit of the full model (apart from population size and growth rates, which were always included), and the most parsimonious models were preferred on account of the small size of the sample. Results are presented as odds ratios, a measure that expresses how much the explanatory variable increased or decreased the probability that a town experienced high cholera mortality, relative to an odds ratio of one (i.e. no effect). Odds ratios above one indicate that higher values of the variable were associated with high cholera mortality (≥1 death/1,000 population), and values below one were associated with lower cholera mortality.

**Table 3.** Logistic regression models of the odds of high crude cholera mortality (1/1,000 or higher) in 1848–49.

Explanatory variable	Bivariate models		Model 1 Transport		Model 2 Transport, topography		Model 3 Full model		Sample mean (S.E.)^a^
	Odds ratio	CI	Odds ratio	CI	Odds ratio	CI	Odds ratio	CI	
(ln) population 1851	1.54**	1.18–2.02	1.47*	1.07–2.02	1.40*	1.01–1.92	1.60*	1.13–2.28	9.4 (0.90)
(ln) annual growth rate 1811–1851	1.16	0.83–1.62	0.98	0.67–1.45	0.95	0.64–1.41	0.95	0.63–1.43	0.6 (0.69)
Turnpike	0.88	0.31–2.50	N.S.						95.1 %
Railway station	0.92	0.59–1.44	N.S.						51.5 %
Canal	1.43	0.91–2.24	N.S.						42.0 %
Seaport	4.33***	2.48–7.55	4.79***	2.64–8.72					24.8 %
Navigable river	3.11***	1.80–5.36	2.25**	1.25–4.06	2.60**	1.39–4.87	2.36**	1.25–4.44	24.1 %
Tidal waterway	4.24***	2.39–7.53			N.S.				23.1 %
Seaport*tidal									
Tidal not seaport					3.60*	1.11–11.65	3.16	0.96–10.34	4.6 %
Tidal and seaport					4.88***	2.44–9.76	4.55***	2.26– 9.17	18.6 %
Seaport not tidal					11.18***	3.40–36.70	10.21***	3.09–33.72	6.2 %
On a river	1.11	0.51–2.42			N.S.				91.2 %
Prone to flooding	1.48	0.87–2.54			N.S.				25.8 %
(ln) altitude	0.54***	0.42–0.69			N.S.				3.8 (1.0)
(ln) ruggedness	0.95	0.71–1.28			N.S.				2.2 (0.8)
bedrock types	N.S.				N.S.				
Coal-bearing strata	1.03	0.65–1.64			1.75	1.00–3.08	1.99*	1.11–3.55	35.2
Mining town^c^	5.40*	1.13–25.83					N.S.		2.6 %
Textile town^b^	0.41*	0.18– 0.90					0.36*	0.14–0.94	11.4 %
Intercept			0.01**	0.00–0.22	0.01**	0.00–0.25	0.00**	0.00–0.10	
Pseudo-r-squared			0.13		0.15		0.16		
Log likelihood ratio^d^			38.30 (*p* < 0.000, 2 d.f.)		49.95 (*p* < 0.000, 3 d.f.)		53.13 (*p* < 0.000, 4 d.f.)		
N	307		307		307		307		

Notes: *p* < 0.05 (*), *p* < 0.01 (**), *p* < 0.005 (***). Variables with *p *≥ 0.05 were included if their inclusion significantly improved model fit.

^a^Values represent either mean and standard deviation, or the percentage of towns with binary value = 1.

^b^Northern and midland towns designated ‘textile’ were: Accrington, Ashton under Lyne, Barnsley, Blackburn, Bolton, Bradford, Burnley, Bury, Carlisle, Chorley, Coventry, Darwen, Glossop, Halifax, Heckmondwyke, Heywood, Hinckley, Hindley, Huddersfield, Hyde, Keighley, Kidderminster, Leeds, Leigh, Macclesfield, Manchester, Morley, Oldham, Preston, Radcliffe, Rochdale, Stockport, Tyldesley, Walton le Dale, Wigan.

^c^Mining towns in sample were: Allendale, Bilston, Dudley, Holywell, Llanelly, Merthyr Tydfil, Oldbury, Tipton, West Bromwich, Wolverhampton.

^d^Relative to model including only (logged) population and growth rates.

If northern industrial and manufacturing towns were at particular risk of cholera in the first half of the nineteenth century then we would expect that rapid population growth, but not necessarily size, would be associated with higher risk of a major cholera outbreak. However rapid growth rate was not associated with the probability of a severe cholera epidemic in either bivariate or multivariate analyses (Table 3). Absolute population size was however positively correlated with higher cholera risk in all models. With the exception of Birmingham all the major towns (population ≥100,000 in 1851) experienced crude cholera deaths rates of over 1/1,000 population, in contrast to the wide range of values in smaller towns.^14^ Therefore larger populations were at higher risk of significant cholera mortality, although the case of Birmingham also indicates that it was possible even for relatively large towns to avoid this fate.^15^

We included measures of transport connectedness in our multivariate models to capture the risk associated with import of cases, both in sparking epidemics, and in contributing to the death toll. However towns with turnpike roads, railway stations and canals were not at higher risk than towns without these features in our models. This apparent unimportance of transport connections was not simply because larger towns were more likely to be better connected, because the lack of association was evident in bivariate analyses (where population size was not adjusted for), as well as multivariate models.

Seaports and navigable rivers were, however, strongly associated with higher cholera mortality in both bivariate and multivariate models. Contemporaries attributed the lethality of seaports both to their openness to the introduction of disease, and their frequent location on tidal rivers, where the sewage of the town was washed back up the watercourse on the tide (Farr, 1868, p. lvi). While seaports were open to international importation, the general lack of association between high cholera mortality and other measures of connectedness apart from navigable rivers suggested that connectedness per se was not an important factor. When we included location on a tidal river in our multivariate models, then this variable was not significantly associated with high cholera mortality, however when we included an interaction between seaport and tidal river variables, then being on a tidal river raised the risk of high cholera mortality for towns that were not ports, compared with towns that were neither ports nor on tidal rivers (OR = 3.60, *p* = 0.033, model 2). Ports on tidal rivers were also at higher risk (OR = 4.88, *p* < 0.000); however the highest relative risk was associated with seaports that were not on tidal rivers (OR = 11.18, *p* < 0.000). These were relatively rare, and included a mix of ports on bays and estuaries (e.g. Folkestone, Gravesend, Holyhead and Swansea), and ports at the bottom of relatively steep valleys (e.g. Hastings). The causes of such high risk of large outbreaks in these types of ports require further investigation. However it does suggest that the higher risk of significant cholera mortality in seaports was not primarily a function of their frequent location on tidal rivers, although tidal rivers also posed a health challenge to towns.

In our model rivers *per se* were not associated with heightened risk in either bivariate or multivariate models, despite the probability that rivers were used both as sources of drinking water and as sewage receptacles (Farr, 1868; Snow, 1855). However *navigable* rivers were associated with higher cholera mortality, and these were more prone than other watercourses to pollution for several reasons. First, navigable rivers were generally larger and slower-flowing than non-navigable waterways, and therefore their relative lethality may reflect the slower removal of wastes from the local area, or the greater accumulation of waste from settlements upstream, compared with smaller faster-flowing watercourses deriving from more local sources (such as the fast-flowing streams flowing out the Pennines, that watered many of the textile towns). Second, navigable rivers may also have been more prone to contamination because of the type of transport involved. Snow alluded in passing to the potential importance of boat traffic in contaminating rivers (Snow, 1855, p. 98), and it is probable that boat-dwellers were more likely than other travellers to catch cholera from contact with infected river or canal water, and to disseminate any infection because they deposited their sewage directly into the watercourse. Canals were liable to the same sources of contamination, however canals were only rarely used for water supply. Where they were, as documented in Exeter, Merthy Tydfil and parts of London, then the results seem to have been severe (Hardy, 1991, p. 80).

We attempted to capture some of the variation in natural sources of water using variables that described the local geology of urban areas. Before the widespread acceptance of the water-borne nature of cholera infection, telluric theories of the origins of cholera were common, based on observed associations between cholera and particular geological features (Pelling, 1978, p. 148). We found no statistical associations between our categories of bedrock types, or of groundwater availability, except in the case of rocks associated with coal deposits, where inclusion of this variable improved the model fit but the odds ratio was not significant (Table 3 model 2: discussed further below). The general lack of correspondence between hydrogeological variables and cholera mortality could reflect the inadequacy of the variables we constructed to proxy natural water sources. However, it also highlighted the complex issue of the sources of water that urban populations depended upon. While rural populations could be considered to rely mainly on very local sources, urban populations often took advantage of water supplies at a greater distance, supplied by conduits or pipes, and therefore measures of the local geology of towns did not necessarily capture the quality of even ‘unimproved’ urban water supplies.

Other topographical variables designed to capture risk of contamination of wells (whether 50% or more of the town area was prone to flooding), and natural drainage potential (altitude and ruggedness, or variance in altitude within each town) were not significantly associated with cholera mortality in our multivariate model (Table 3, model 2). The importance that contemporary commentators attributed to altitude was evident in bivariate analysis, but disappeared once the effects of seaports and navigable and tidal rivers were included.

Location on coal-bearing strata was not associated with higher risk of significant cholera mortality in bivariate models, but improved model fit once the effects of population size and growth, seaports and navigable rivers were adjusted for (OR = 1.75, *p* = 0.051, model 2). To test whether this association was a function of the higher risks associated with mining districts we included a variable indicating towns with a significant involvement in mining.

Mining towns were some of the worst affected by cholera. Mining towns grew up very rapidly in this period, and their vulnerability to cholera outbreaks suggests superficially a connection to rapid and unregulated growth of the kind posited more widely for industrialising towns. The mines themselves lacked toilet facilities, so miners were easily infected at work (Snow, 1855, p. 19,111), and water drained from the mines, and then sometimes supplied by the mining company to the local population, was also liable to contamination. Another potentially lethal aspect of mining towns was the effect of the mining operations themselves on pre-existing water sources. Mines required regular draining to prevent flooding, and such drainage could affect local water tables well beyond the mined area, drying up longstanding sources of water supply and forcing the inhabitants to resort to less salubrious sources.

However although the high risk of mining towns was evident in bivariate analysis, the association with high cholera mortality weakened when other variables were included (Table 3).^16^ This suggested therefore that the association between coal-bearing bedrock and high cholera mortality was not directly related to mining, and may instead have reflected an elevated risk associated with towns more generally in these areas. The bedrock types associated with high cholera mortality comprised a swathe running from the Northumbrian coalfields in the north-east of England through eastern Lancashire and the West Riding of Yorkshire, plus outcrops including the Black Country near Birmingham, and the coalfields of south Wales. The dependence of nineteenth-century industry on proximity to coal, before the development of railways, meant that not only mining towns but many of the industrial and manufacturing towns that developed in the nineteenth century were located on or near these coal-bearing strata.

In contrast to mining towns, many of the northern textile mill towns were notable for their relatively low mortality in successive cholera epidemics (Figure 5(b,c), 7). Textile industry was particularly strongly associated with rapid population growth, but was associated with low cholera mortality in both bivariate and multivariate analyses (OR = 0.36, *p* = 0.037, model 3; Figure 7(c,d)). Many of the northern textile towns were sited to take advantage of fast-flowing (non-navigable) streams for mechanical power, and these were drawn from relatively pristine local watersheds in the Pennines. In addition, some of these textile towns were supplied with upland waters piped under a continuous system (that is, always available, not intermittently supplied).^17^ While the willingness to supply relatively cheap and clean piped water was doubtless motivated partly by the requirements of the textile industry for soft clean water, it also reflected the basic geographical advantages of these towns. That is, the local sources of both piped and ‘unimproved’ water were relatively clean (Durey, 1979, pp. 43–45). Most of these towns were located on or near coal measures, and adjusting for their lower cholera mortality in the model increased the size and statistical significance of the odds that towns on coal-bearing strata experienced higher mortality (OR = 1.99, *p* = 0.020, Table 3).

Importantly, our models could not capture some of the most important sources of variation in our sample. First, there was an inherently stochastic element to the occurrence of a cholera outbreak, because it depended on a chain of events such that even if cholera was introduced successfully into a settlement or a water supply, it did not necessarily result in reported deaths. By considering only towns of population 5,000 or more we reduced the probability that cholera was never introduced, but we could not assume a consistent case-fatality rate. Second and more importantly, we had no direct information on water sources or quality, and how these might have changed between epidemics. For example, Snow cited the case of Hull, where the town was supplied only very scantily from springs in 1832, and suffered moderate cholera mortality. Around 1844 the water supply was augmented with a new waterworks drawing its supply from the river Hull, a tidal river into which the town’s sewage also flowed, and the cholera mortality of 1849 was severe although ‘the town was much better drained in 1849 than in 1832ʹ (Snow, 1855). Our topographical, demographic and economic variables could not capture this most crucial type of information directly, regarding water provision and sanitation.

Taken together, our analyses and the evidence from Figure 5–7 serve to confirm the impression given by successive reports of cholera in Britain, that cholera mortality was not particularly focussed on northern industrialising or manufacturing towns but displayed a very widespread extent with several major areas of high mortality, or what Farr described as ‘cholera fields’. Farr’s cholera fields for 1849 included London, Portsmouth, Plymouth, Bristol, Merthyr Tydfil, Wolverhampton, Liverpool, Hull and Tynemouth, a fairly catholic group of districts which excluded most of the major northern manufacturing areas of Lancashire and the West Riding of Yorkshire. While mining districts were identified by contemporaries as particularly prone to cholera outbreaks, manufacturing towns were not singled out as at peculiar risk.^18^ This rather even-handed attitude is consistent with the tone of the Health of Towns reports of 1840 and 1844–1845, and Chadwick’s 1842*Report on the sanitary condition of the labouring population*. With respect to water supply, the authors of the Health of Towns reports did not single out northern or manufacturing towns as especially deficient (Dennis, 1984, pp. 20–23). Instead Bristol, which Szreter and Mooney identified as exemplifying a long-established city with relatively high life expectancy in the 1840s, was described in the second Health of Towns report as having the most inadequate water supply of any town of comparable size in England, lacking any statutory provision (Parliamentary Papers, 1844, p. 89). As Durey notes, when Chadwick sought to exemplify the most egregious cases of deficient water supplies, he resorted almost entirely to Scottish examples (Chadwick, 1842, pp. 135–142; Durey, 1979, pp. 44–47).

The evidence from the cholera epidemics of 1832 and 1849 suggests that the presumption that older, long-established towns coped better with urban growth than newer or more industrial towns does not hold with respect to water quality. The rate of urban growth was unimportant with respect to cholera mortality, but the size of towns and their position as seaports or on navigable rivers exposed them to higher risk. In addition we identified a doubling of risk associated with location on coal-bearing strata, once the lower mortality of textile towns was adjusted for. However the archetypal towns of the industrial revolution, the textile mill towns, and the manufacturing city of Birmingham were distinguished by very modest cholera mortality, while many of the older, slow-growing southern towns, and London, were characterised by much more lethal cholera outbreaks. London had the most extensive water supply and sewerage system of any town in England, but cholera mortality was promoted there by the delivery of contaminated water to thousands of households.

If we accept that cholera mortality does provide a measure of the extent of sewage contamination of domestic water supplies, then it would appear that the quality of water supplies improved after 1850, rather earlier than usually assumed. The low mortality of the 1866 epidemic, and the subsequent failure of cholera to re-establish itself in Britain, despite continued epidemics on the Continent and in the United States, points to the success of several strategies adopted by the British government and local health authorities after 1854. First, quarantine, which was resisted by mercantile interests in the first three outbreaks, was enforced more strictly and effectively in 1866 and during succeeding scares (Hardy, 1993). This clearly did not prevent cholera from entering Britain and spreading in 1866, but it probably reduced the multiplicity of sources and volume of introductions.^19^ Secondly, central directives regarding the isolation of cholera victims, disposal of contaminated bedding and clothing, and decontamination of dwellings were enforced more strictly with growing experience of successive epidemics. The growing recognition, after 1854, that water was implicated in the spread of cholera was probably also important in prompting the temporary closure of water sources thought to be implicated in outbreaks, although this policy was very patchy (Luckin, 1977). Finally, and possibly most importantly (given the evidence of extensive spread but low mortality in 1866), many towns improved and overhauled their water supplies before 1866 (Hassan, 1985; 9th Report of the Privy Council, 1867). Hassan has demonstrated that it was the larger industrial towns which tended to municipalise and modernise their water supplies first, with substantial municipal acquisitions in the period between 1841 and 1860 (Hassan, 1985, p. 536, 540). While major improvements in the quality of water supplies are usually considered to have been achieved only in the period of extensive municipalisation after c.1870, the relatively low mortality of the 1854 and 1866 epidemics and the precocious disappearance of cholera from Britain after 1866, together with the precipitous decline in dysentery after 1850 (Figure 2), supports contemporary perceptions that provision of piped water from relatively uncontaminated sources was effective in reducing urban mortality, especially from typhoid, the main endemic water-borne disease (9th Report of the Privy Council, 1867).^20^

Does the finding that cholera mortality was not especially focussed in rapidly growing manufacturing and industrial towns mean that these towns were more generally unexceptional at least with respect to gastro-intestinal infections? Probably not. While we have argued that cholera was primarily waterborne and therefore provides a good indicator of faecal contamination of domestic water supplies, water was not the only source of gastrointestinal pathogens. In the final section we discuss the persistence of high rates of infantile diarrhoea in northern and midlands towns, and the relationship between diarrhoeal and cholera mortality. We address in particular the question, if water supplies were not especially deficient in these towns, then why did they experience such high diarrhoeal mortality in the second half of the nineteenth century?

## 4. Cholera mortality and the ‘sanitary-diarrhoeal’ complex in urban centres

‘The rise of new manufacturing towns, with the great extension of the borders of old towns, as in Lancashire and Yorkshire, has inevitably brought to the fore this distinctive fatality of infants [infantile diarrhoea]. … It is remarkable however that there are only a few of them, such as Liverpool and Hull, that have been the chosen seats of great epidemics of Asiatic cholera.’ (Creighton, 1894, pp. 762–763).

Writing at the end of the nineteenth century, Creighton was well aware of the persistent toll of infantile diarrhoea in urban centres, a mortality that was especially marked in manufacturing towns and ‘certain seaports’ (Creighton, 1894, p. 761). While life expectancy rose in the English population from the 1870s, infant mortality did not improve until after 1900, and the major cause of these persistently high infant mortality rates was diarrhoeal mortality in urban populations (Woods, Watterson, & Woodward, 1988). The English towns with the highest rates of diarrhoeal mortality were almost all northern and midlands manufacturing and industrial towns, that had experienced rapid expansion in the nineteenth century (Table 4) (Woods, 2000, pp. 328–331). Yet, as Creighton remarked, these towns were not distinguished by high cholera mortality.

**Table 4.** League table of diarrhoeal and infant mortality rates in English cities, 1871–80.

Town	Diarrhoeal mortality, under fives (per 1,000)	Infant mortality (deaths in first year of life/1,000 births)
**Leicester**	17.81	214
**Preston**	15.61	212
Yarmouth	14.38	199
**Liverpool**	14.13	217
**Salford**	12.44	184
**Leeds**	12.02	188
**Birmingham**	11.78	179
**Sculcoates**	11.64	170
**Manchester**	11.23	190
**Wigan**	11.13	172
**Worcester**	11.10	176
**Hull**	11.02	178
**Sheffield**	10.96	183
**Holbeck**	10.93	196
Northampton	10.85	173
**Hunslet**	10.75	192
**Coventry**	10.06	164
**Stoke-on-Trent**	9.91	189
**Nottingham**	9.86	184
Norwich	9.78	188
**Hartlepool**	9.43	166
London St Giles	9.42	176
London Whitechapel	9.24	181
**Goole**	9.20	166
**Bolton**	9.13	167
**Blackburn**	9.02	191
**Stockport**	9.05	182

*Source*: (Creighton, 1894, p. 76)

*Notes*: towns in bold are located in northern or midlands counties.

Levels of infant diarrhoeal mortality have often been used as a measure of the sanitary conditions, and explicitly or implicitly, water quality in urban settlements. If the latter assumption was correct then the disparity between cholera and diarrhoeal rates in northern towns would suggest that cholera mortality was a poor measure of water quality.^21^ An alternative explanation for the disparity is that infant diarrhoeal mortality was not primarily a function of water quality, a conclusion reached by most contemporary authorities by the late nineteenth century (Hardy, 2014, Chapter 4; Luckin, 1986, pp. 108–115; Woods, Watterson, & Woodward, 1989).

Several lines of evidence support this second conclusion. First, although contemporary efforts to isolate the causal agents of diarrhoeal mortality were unsuccessful, they were probably pathogens of fairly low virulence, such as those clustered at the bottom of Table 1 (Hardy, 2014, pp. 71–74; Martin, 1913). These pathogens were associated with predominantly person-to-person or food-borne outbreaks. Table 1 demonstrated an empirical association between virulence and waterborne transmission. Ewald’s evolutionary explanation for this relationship relates the level of virulence of pathogens to their mode of transmission (Ewald, 1991, 2004). Briefly, for pathogens that rely on direct person to person transmission for propagation, then there is strong selection for avirulence, since drastic effects on the host that reduce the host’s mobility reduce the chances of transfer of the pathogen to the next host. However for pathogens that rely on other modes of transmission there is less selection against virulence. In the case of cholera, the pathogen releases toxins that cause massive water loss from intestinal cells, causing very rapid dehydration and excretion of large volumes of fluids containing the cholera bacterium. The victim was usually rendered prone and extremely ill, but the contaminated watery excretions were carried away in sewage, or when contaminated clothes and bedding were washed. Moreover free-living cholera bacteria can survive and multiply in fresh or saline water, amplifying their capacity for transmission. By contrast faecal-oral pathogens with low virulence may rely on transfer from infected but ambulatory victims during food or drink preparation or person to person contact. The low virulence of diarrhoeal pathogens is suggested by the concentration of diarrhoeal mortality at the extremes of life, in comparison with cholera (Figure 3). That is, although all ages are liable to diarrhoeal infections, these infections are usually only lethal to very small children (who dehydrate easily, are immunologically naïve, and who lack the reserves to endure prolonged dehydration) and those with co-morbidities, including the elderly. If Ewald’s evolutionary hypothesis is correct, then the low virulence of diarrhoeal diseases would suggest that the latter types of routes were most important.

Second, as discussed above, diarrhoea mortality did not follow the same trajectory of decline as the more water-dependent diseases, cholera, dysentery and typhoid (Figure 2).

However, if we accept that infantile diarrhoea was not a function of water quality, a puzzle remains. Infants may have been subject to diarrhoeal infections that were lethal to them and not to older children and adults, and that persisted in intensity across the nineteenth century. However they should still have benefitted from reductions in exposure to waterborne infectious diseases, benefits that should be reflected in declines in either diarrhoeal or all-cause infant mortality. Yet there was no improvement in either rate, at the national level, until 1900. Again, cholera mortality may provide a partial answer to this question.

**Figure 8.**
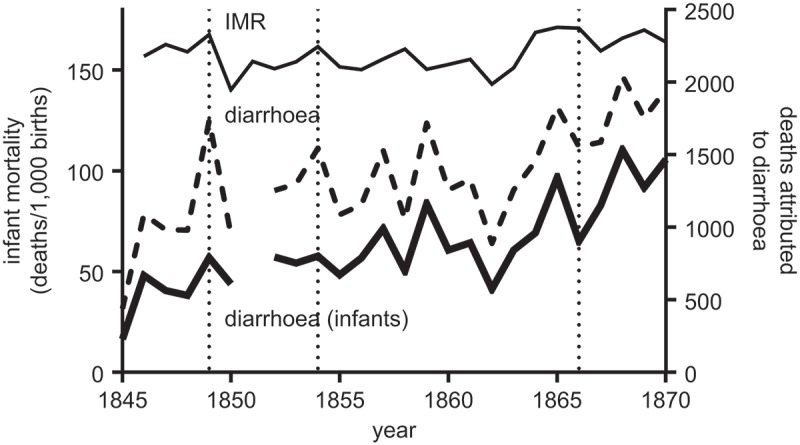
Annual infant mortality in London, and deaths attributed to diarrhoea in infancy (dashed lines) and at all ages (heavy solid line). Dashed vertical lines indicate years of cholera epidemics. Sources: Annual Reports of the Registrar-General (1845–1870).

Figure 3 suggested that infants experienced lower cholera mortality than older children. An obvious explanation is that deaths due to cholera were under-reported in infants, perhaps because they died before the characteristic symptoms manifested (Farr, 1868, p. xli). We can test indirectly whether cholera mortality was under-reported in infants, or reported under diarrhoeal deaths, by comparing the effects of cholera epidemics on all-cause mortality in infants, and diarrhoeal mortality, in London, where we have annual series (Figure 8). Surprisingly, neither infant all-cause nor infant diarrhoeal mortality rose notably during the cholera epidemics of 1849, 1854 or 1866, despite the large impact of cholera on London in each of these years (see also Luckin, 1986, pp. 106–108).

**Figure 9.**
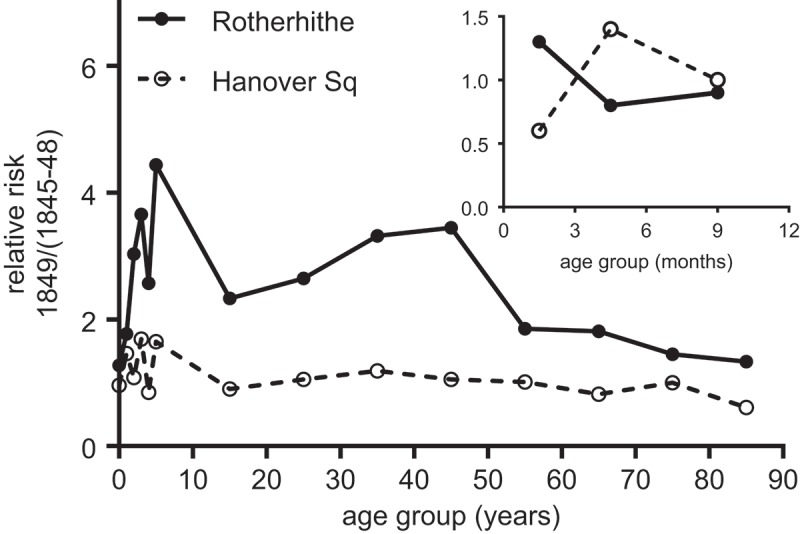
Risk of death by age group for males in the year 1849 relative to years 1845–1848, in the registration districts of Rotherhithe and St George, Hanover Square, London. Sources: Annual Reports of the Registrar-General (1845–1849)

A further more discriminating test of the impact of cholera on infants is presented in Figure 9. Here we compare the impact of the 1849 cholera epidemic in Rotherhithe, the London district with the highest cholera mortality in 1849 (crude cholera death rate in 1849 20.5 deaths/1,000 population) and in St George Hanover, the district with the lowest (crude cholera death rate of 0.8/1,000). In Hanover Square the age-specific risk of dying in 1849 was very similar to the previous four years (relative risk was close to one for all ages except young children). However in Rotherhithe the crude death rate doubled in 1849 relative to the previous four years, and the relative risk in 1849 was between 1.5 and 4.5 for all ages except infants (i.e. between 1.5 and 4.5 times higher in 1849 that in 1845–1848). This increased risk was almost entirely attributable to the ferocity of the 1849 cholera outbreak in Rotherhithe. Notably however, relative risk in 1849 was lower at the extremes of age, especially in infancy. These are the age groups where mortality is higher in any case, and so it was possible that significant cholera mortality in these age groups was masked by its relatively small additive effect on total death rates. To test this possibility we examined mortality within the first year of life (Figure 9, inset). In infants diarrhoeal mortality was usually highly concentrated in the second half of the first year of life, because younger infants were protected to a significant extent from gastro-intestinal diseases by breast-feeding and by their own immobility that reduced environmental exposures. Importantly, there was no rise in risk in 1849 amongst older infants in Rotherhithe. Moreover mortality rates at ages 6–11 months were virtually the same in Rotherhithe and Hanover Square in 1849 (45.3 and 45.1 respectively), despite the explosive cholera epidemic that claimed 800 lives in the former district. These results strongly suggest that infants were indeed relatively immune to cholera infection.

**Figure 10.**
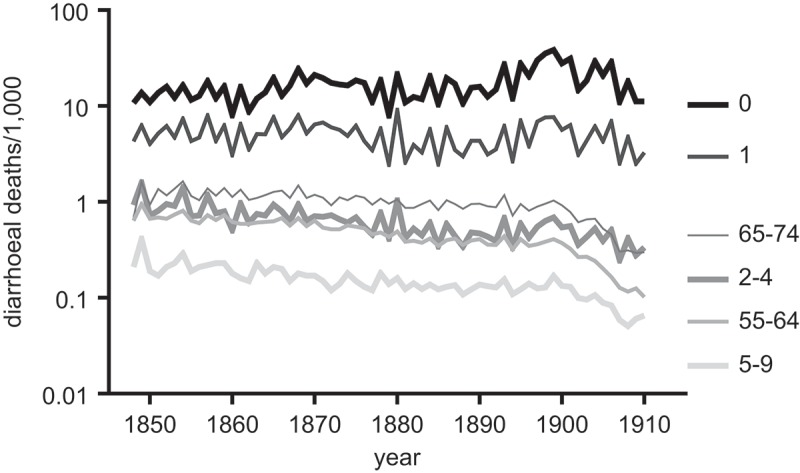
Annual age-specific death rates attributed to diarrhoea, England and Wales. *Notes*: Deaths attributed to diarrhoea were reported separately 1848–1880, and combined with dysentery 1881–1911 (when dysentery rates had fallen to negligible levels – see Figure 2).*Sources*: Davenport (2007).

Finally, although diarrhoeal mortality remained high for infants and very young children until the end of the century, diarrhoeal mortality rates fell for older age groups (Figure 10). The lack of improvement for infants and one-year olds suggests again that the causal agent of diarrhoea in these age groups, or the mode of transmission, differed in these age groups compared with older children and adults (see also Woods, 2000, pp. 330–331).

Taken together these lines of evidence suggest that infants were relatively immune to water-dependent diseases, which implies that they were not exposed to any significant extent to contaminated water, but were infected with diarrhoeal diseases via other routes. We cannot examine here what this implies about infant feeding practices, except to note that breastfeeding practices cannot account entirely for this phenomenon. Not all infants were breastfed, and many were only partially breastfed or weaned in the first year of life. Therefore there must have been other infant feeding practices, such as the use of boiled water or the avoidance of water that accounted for the low susceptibility to cholera of very young children (Woods, 2000, p. 331). Similarly, we can only speculate about the roles of fly-borne and food-borne transmission in infant diarrhoeal diseases.

## 5. Conclusion

We analysed the geographical patterning and severity of cholera epidemics in nineteenth century England, in order to test the argument that waterborne diseases were especially rampant in rapidly growing manufacturing and industrial towns. We found that there was no obvious geography to cholera mortality that would support this argument. Our regression analyses identified many of the same factors isolated by contemporary observers, including population size, seaport status, and location on tidal or navigable rivers or on coal-bearing strata. The latter was associated with many northern industrialising towns, as well as mining towns. However northern textile towns were at notably low risk. We speculated that the relative freedom from cholera of Birmingham and the textile towns of Lancashire probably reflected the natural advantages of these places with respect to clean sources of water (fast-flowing streams and abundant springs), and possibly the advantages of tardy development with respect to sewerage. Conversely, investments in drainage and water supply did not necessarily reduce, and could aggravate, the risk of cholera, as the case of London demonstrates.

If we accept the argument that northern manufacturing and industrial towns were not, on average, exceptional with respect to the pollution of water supplies, compared with many older and ostensibly better regulated towns, then did they enjoy similar levels of health and longevity in the period 1830–1850? Probably not. The higher infant and child mortality rates evident in the second half of the nineteenth century in many northern towns was probably also a feature of the first half of the century, as Szreter and Mooney argued. However it seems unlikely that this excess mortality was due to inferior water quality. Our evidence indicates that infants were relatively shielded from infection with water-borne diseases, including cholera. On the other hand, they did not enjoy the falls in mortality from these causes that were, we suggest, associated with progressive improvements in the purity of drinking water at least after c.1850, and this must explain to some extent the anomalous persistence of high rates of infant mortality as survival improved amongst older children and young adults.

Did water quality deteriorate, on average, in urban centres during the rapid urbanisation of the period 1800–50? Clearly mining developments, exhaustion or pollution of local water supplies, flush toilets, and increased sewerage release into watercourses increased the risk of faecal contamination of water supplies in specific cases. In this sense, Szreter and Mooney are probably correct in assuming a net decline in water quality, that could have been associated with rises in rates of waterborne diseases. However there was little evidence in our study that this effect was confined to ‘new’, northern, or manufacturing and industrial towns. Moreover some of these developments, especially the increased release of sewage into rivers, should probably not be considered as the consequences of unplanned growth, but rather as nascent attempts to deal with the consequences of rising urban populations, albeit in a manner that could operate to worsen mortality, and to increase inequalities in health.

It also seems plausible that water quality improved between 1849 and 1866. Many towns ‘improved’ their drainage and water supply systems between 1832 and 1866, sometimes with disastrous results (as in the case of Hull, cited above). However the reduction in cholera mortality after 1849 and its disappearance after 1866 raises the possibility of significant improvements in average water quality well before the onset of major municipalisation of water companies from c.1870. Crucial to this argument is the recognition that relatively crude interventions may have had disproportionately large impacts on gastrointestinal disease mortality because they reduced transmission of the most lethal diseases. The introduction of unfiltered upland water supplies, or crudely filtered non-tidal river water, may have been sufficiently to reduce radically the risk of outbreaks of cholera, and water-borne typhoid and dysentery. To make further progress in eliminating remaining waterborne outbreaks, and in reducing mortality due to food, person-to-person and fly-borne disease transmission, would then have required much greater investment in a range of interventions. These include more stringent controls on water quality; the provision of greater volumes of clean water to improve domestic hygiene and reduce transmission via food- and person-to-person routes; and major investments in sewage disposal. However these investments probably yielded diminishing returns, once the low-hanging fruits (the most virulent and easily preventable diseases) had been picked.

Finally, infant diarrhoeal mortality was a major cause of the urban penalty in British cities, and there is currently no reason to think that this element of mortality worsened in the period 1820–50. This assessment is based on the insensitivity of infant diarrhoea to improvements in sanitation and water supplies after 1850, which implies a similar insensitivity to any worsening of these factors before 1850. Infant mortality and diarrhoeal mortality rates were also insensitive to population density, above a rather low threshold, and again this implies that these types of mortality were relatively insensitive to increases in urban crowding and associated disamenities (Woods, 2000, pp. 328–329). However in the absence of evidence of the underlying causes of infant diarrhoeal mortality we cannot yet assess this question. What does seem clear however that attempts to ameliorate the water problems of Victorian cities benefited mainly older children and adults, with little benefit to infants.

A key finding of this study was the apparent exemption of infants from high cholera mortality. That infants were relatively protected from waterborne cholera, and conversely very susceptible to non-waterborne gastro-intestinal diseases, has two major implications. First, the impact of water quality and waterborne diseases on young children may vary dramatically between societies and sub-populations, depending on how cultures of infant care affect exposure to water, independent of water quality. Diversity in exposure of infants to contaminated water may therefore explain some of the differences between studies in the impact of improved water on outcomes for infants. Secondly, large improvements in water supply and/or sanitation (which also improved markedly in the last quarter of the nineteenth century) may have a negligible impact on infant or diarrhoeal mortality, but large effects on mortality in other age groups or from other causes, such as typhoid, dysentery or cholera, raising important issues with respect to the choice of outcomes used to evaluate the effectiveness of interventions.

## Notes

1. Liverpool, England’s second city, had a population of only c.78,000 in 1801.

2. It also seems likely that as water-related outbreaks of typhoid diminished food-borne transmission came to dominate, and domestic hygiene became a more important factor (Hardy, 1993, p. 159, pp. 161–165; Luckin, 1986, pp. 134–135). This may explain the slower rate of reduction in typhoid mortality after the mid-1880s, and the resumption of improvements in tandem with improving diarrhoeal mortality after c.1900.

3. Dysentery was probably mainly waterborne, and mortality rates were reported 1848–80 for the country as a whole, but were not reported as a separate category below the national level.

4. The choice of threshold is discussed in ‘Data sources and methodology’, below.

5. Typhoid was also sufficiently lethal to kill young adults, and the celebrated deaths and near-fatalities of adult members of the Royal family (including Queen Victoria’s husband Albert) and aristocracy from typhoid may have been even more influential than cholera epidemics in spurring local authorities to improve water supplies.

6. National Archives, PC1/108, *Manuscript as to the incidence of cholera in Great Britain*.

7. Each place was numbered in order of date of outbreak, and the numbers originally referred to a map, but the map does not appear to have survived. National Archives, ‘Manuscript as to the incidence of cholera in Great Britain’, PC1/108.

8. This dataset was compiled from the work of Law and Robson, among others, and includes their census populations for urban units of population 2,500 or more. The urban units comprise census units of various types: the population within the legal limits of a municipal or parliamentary borough for towns constituted as boroughs; otherwise the parish in which the town was located; or the parts of parishes comprising the unit regarded by county census officials as comprising a ‘town’. Where town populations were missing from the dataset then we extracted the population from the 1851 census.

9. Law & Robson did not provide populations for towns below their urban threshold of 2,500. However Clark and colleagues supplied population counts for small English towns in 1811 and 1851. For Welsh towns, and for places in our dataset that were not considered towns in 1811, we extracted population totals for the smallest units reported in the 1811 census.

10. Evident in the diagonal patterns created especially in Figure 7(b) by the lower bound on crude death rates imposed by very small numbers of deaths in small populations.

11. We used a 500 metre buffer, rather than for instance a requirement that the town footprint and the transport feature intersected, because the town footprints delineated the built-up area, and therefore sometimes excluded substantial waterways within the town limits, or a railway station, if it was located on the outskirts of the town.

12. This dataset included only 160 English and 23 Welsh towns and therefore could not be used to identify the full 307 towns in our sample as manufacturing or industrial based on the share of secondary employment. However this dataset included all mining and textile towns in our sample that were described as such in more qualitative sources.

13. As noted in the spring of 1866, ‘[E]vidently England was being infected in many different directions, and could hardly hope to escape a serious extension of the disease’ (Medical Officer of the Privy Council, 1867, p. 19).

14. Note though that sensitivity analyses indicated that other choices of cut-off levels of crude cholera mortality to create the binary outcome variable did not produce markedly different model parameters.

15. The apparent contradiction between this finding and Figure 7(a,b) is due to the use of a binary definition of outbreak size in the regression model. Larger towns were more likely than smaller towns to experience an outbreak with mortality of at least 1 death/1,000. However small towns were more vulnerable to extreme rates, not simply as a function of stochasticity, but because they were more likely to rely on a small number of water sources.

16. This was the case whether the variable indicating coal-rich strata was included or excluded from models.

17. The Lancashire textile towns of Ashton under Lyne, Bury, Oldham and Preston were singled out for praise in the second State of Large Towns report (Royal Commission for inquiring into the state of large towns and populous districts, 1845, pp. 400–414), and Snow used Preston and Oldbury as examples of the merits of upland sources of piped water (Snow, 1855, p. 102).

18. Farr noted the high mortality of the ‘principal districts’ on the coal fields, but the districts he singled out were those most strongly associated with mining operations (Farr, 1852, p. lvii; Baly & Gull, 1854, p. 7). It is possible that the positive association we found between coal-bearing strata and cholera mortality, and the lack of association with mining per se, reflected an under-identification of mining-affected areas in our sample.

19. Similarly, quarantines imposed in 1892–1893 in the face of continental outbreaks undoubtedly restricted severely the entry of cholera into Britain, but did not altogether prevent it. Cholera cases were reported at 64 localities, but all outbreaks were very small, indicating that local transmission was prevented by additional factors (Local Government Board, 1894, p. ix).

20. While typhoid mortality was not reported separately from typhus before 1869, mortality attributed to dysentery, another mainly waterborne disease, fell steeply between 1850 and 1880 (when it fell to very negligible levels and was no longer reported separately) (Figure 2). This trend is consistent with an effect of improving water quality after 1850.

21. Or that water quality was good before c.1867, when cholera disappeared, but worsened afterwards. However diarrhoeal mortality was fairly consistent in the decades 1850–1900.
